# Early treatment of minocycline alleviates white matter and cognitive impairments after chronic cerebral hypoperfusion

**DOI:** 10.1038/srep12079

**Published:** 2015-07-15

**Authors:** Jing Ma, Jing Zhang, Wei Wei Hou, Xiao Hua Wu, Ru Jia Liao, Ying Chen, Zhe Wang, Xiang Nan Zhang, Li San Zhang, Yu Dong Zhou, Zhong Chen, Wei Wei Hu

**Affiliations:** 1 Department of Pharmacology, Key Laboratory of Medical Neurobiology of the Ministry of Health of China, School of Basic Medical Sciences, College of Pharmaceutical Sciences, Zhejiang University, Hangzhou, Zhejiang 310058, P. R. China; 2 Collaborative Innovation Center for Diagnosis and Treatment of Infectious Diseases, Hangzhou, Zhejiang 310003, P. R. China; 3 Department of Pharmacy, Xinhua Hospital, Shanghai Jiaotong University School of Medicine, Shanghai 200092, P. R. China; 4 Department of Pharmacy, Sir Run Run Shaw Hospital, School of Medicine, Zhejiang University, 3 East Qingchun Road, Hangzhou, Zhejiang 310016, P. R. China

## Abstract

Subcortical ischemic vascular dementia (SIVD) caused by chronic cerebral hypoperfusion develops with progressive white matter and cognitive impairments, yet no effective therapy is available. We investigated the temporal effects of minocycline on an experimental SIVD exerted by right unilateral common carotid arteries occlusion (rUCCAO). Minocycline treated at the early stage (day 0–3), but not the late stage after rUCCAO (day 4–32) alleviated the white matter and cognitive impairments, and promoted remyelination. The actions of minocycline may not involve the inhibition of microglia activation, based on the effects after the application of a microglial activation inhibitor, macrophage migration inhibitory factor, and co-treatment with lipopolysaccharides. Furthermore, minocycline treatment at the early stage promoted the proliferation of oligodendrocyte progenitor cells (OPCs) in subventricular zone, increased OPC number and alleviated apoptosis of mature oligodendrocytes in white matter. *In vitro*, minocycline promoted OPC proliferation and increased the percentage of OPCs in S and G2/M phases. We provided direct evidence that early treatment is critical for minocycline to alleviate white matter and cognitive impairments after chronic cerebral hypoperfusion, which may be due to its robust effects on OPC proliferation and mature oligodendrocyte loss. So, early therapeutic time window may be crucial for its application in SIVD.

Subcortical ischemic vascular dementia (SIVD) induced by chronic cerebral hypoperfusion as a result of small-artery disease is a common subtype for vascular dementia, which is recognized as the second most prevalent type of dementia^1^. SIVD, including Binswanger’s disease, is often observed in patients with hypertension or atherosclerosis, as well as in the aging population. The characteristic damage of SIVD includes progressive demyelination, white matter rarefaction and cognitive impairments^2^. However, the pathogenetic mechanisms of SIVD after chronic cerebral hypoperfusion are far from understood, with currently no effective treatments to prevent or reverse its progression^3^. Acetylcholinesterase inhibitors and vasodilators are used to treat SIVD but only produce limited benefits^4^,^5^. Therefore, it’s urgently needed to develop new agents for effective treatment or prevention of SIVD.

Minocycline is a semisynthetic second-generation tetracycline and has been used to treat a variety of infectious diseases with relatively few adverse effects^6^. The clinical application of minocycline as an antibiotic became limited due to drug resistance and the availability of more effective agents^7^,^8^. Recently, new functions of minocycline has been discovered, including anti-inflammatory^9^,^10^, glutamate antagonistic^11^, and anti-apoptotic actions^12^. Minocycline can easily penetrate the blood-brain barrier because of its high lipophilicity, suggesting it is an ideal candidate for treating central nervous system diseases. As such, the pharmacological effects of minocycline on several neurological diseases, such as multiple sclerosis^13^, Alzheimer’s disease^14^ and brain injury^15^, have been studied.

Recently, minocycline has been shown to improves neurobehavioral performance, attenuates the hypoxia-induced loss of oligodendrocytes and hypomyelination in the white matter of neonatal rats, possibly by suppressing microglial activation^16^,^17^. Also, minocycline protects against white matter damage by inhibiting microglial activation in rats, which were subjected to bilateral permanent occlusion of the common carotid arteries^18^. However, Tsuji *et al.*^19^ reported that in neonatal mice minocycline displayed a detrimental effect on the white matter after a hypoxic-ischemic insult. In a demyelination model induced by injection of ethidium bromide, minocycline inhibits remyelination by suppressing the production of oligodendrocyte progenitor cells (OPCs) in the lesion area^20^. Thus, it is valuable to reveal the effect of minocycline on SIVD and the underlying mechanism.

Due to the progressive nature of white matter damage after chronic cerebral hypoperfusion in SIVD^21^,^22^, it is intriguing to explore temporal effects of minocycline on white matter damage. Also, the action of minocycline on OPCs and mature oligodendrocytes was investigated in a mouse model of chronic cerebral hypoperfusion established by right unilateral common carotid arteries occlusion (rUCCAO). This model well simulates the behavioral and pathological impairments of SIVD^23^,^24^. We found that the early treatment is critical for minocycline to alleviate white matter and cognitive impairments after rUCCAO. The protection of minocycline may be due to the robust effects of minocycline on OPC proliferation and mature oligodendrocyte loss at the early stage, but not the inhibition of microglia activation.

## Results

### Effect of minocycline on cognitive impairment and white matter damage after rUCCAO

**Figure 1:**
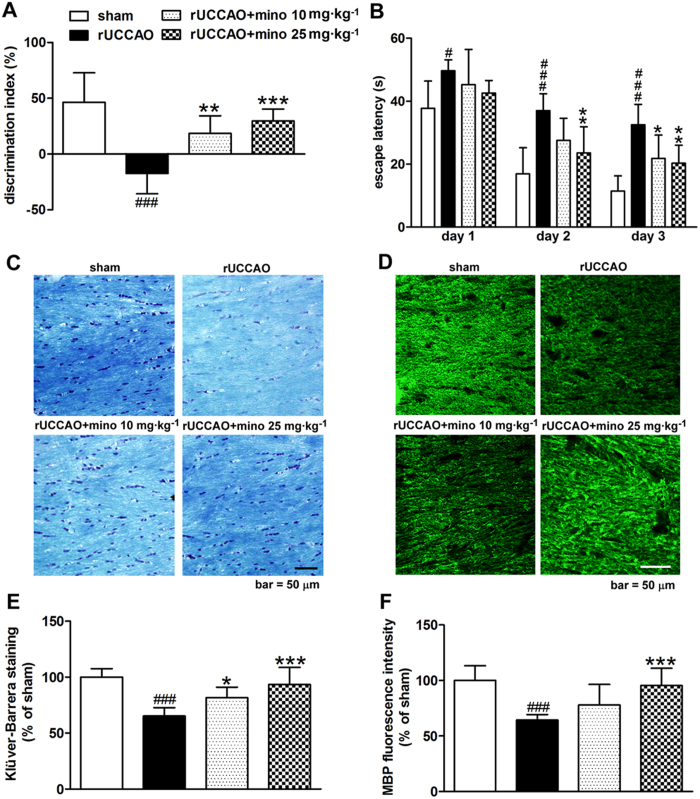
The effect of minocycline (mino) on cognitive impairment was evaluated by the object recognition test (**A**) and the Morris water maze test (**B**) from 27 to 32 days after rUCCAO. The effect of minocycline on white matter damage from the Klüver-Barrera staining (**E**) and MBP expression (**F**) in the corpus callosum were evaluated at 32 days after rUCCAO, with representative photomicrographs in (**C**,**D**). Scale bar, 50 μm. (**A**,**B**) n = 8–9, E, F: n = 6–8. Values are represented as mean ± s.d. #*P* < 0.05, ###*P* < 0.001, *vs*. the sham group; *P < 0.05, **P < 0.01, ***P < 0.001, *vs*. the rUCCAO group.

In this experiment, the cognitive ability was evaluated with both the object recognition test, which examine non-spatial working memory related to the frontal-subcortical circuits^23^,^25^ and the Morris water maze test, which examine spatial memory^23^,^26^. After rUCCAO, mice showed a remarkable decline in discriminative ability in the object recognition test (discrimination index: −17% *vs*. sham group: 46%, *P* < 0.001, Fig. 1A) and prolonged the escape latency in training trials in the Morris water maze test (two-way ANOVA indicated a significant difference, *P* < 0.001, Fig. 1B), which was not observed in the probe trails of the Morris water maze test (Supplementary Fig. S1). When minocycline was administered for the entire process after rUCCAO at a dose of either 10 or 25 mg·kg^−1^d^−1^, it robustly elevated the discrimination index to 18% (*P* < 0.01) and 29% (*P* < 0.001), respectively, in the object recognition test. Minocycline also markedly shortened the escape latency in training trials in the Morris water maze (Fig. 1B). Two-way ANOVA test indicated a significant difference between the rUCCAO group and the minocycline-treated (10 mg·kg^−1^d^−1^, or 25 mg·kg^−1^d^−1^) group (*P* < 0.001).

The white matter damage in the corpus callosum was examined by Klüver-Barrera staining, which can reflect the deficiency of the components of myelin. The intensity in Klüver-Barrera staining after rUCCAO was lower than that after the sham operation (*P* < 0.001, Fig. 1C,E), while it was reversed by minocycline in a dose dependent manner, which almost completely recovered at a dose of 25 mg·kg^−1^ (*P* < 0.001, Fig. 1C,E). Myelin basic protein (MBP) is one of the major family members of myelin proteins, and its expression often markedly declines during demyelination^27^,^28^. We found the decrease of MBP expression was rescued by minocycline at a dose of 25 mg·kg^−1^d^−1^ (*P* < 0.001, Fig. 1D,F). Together, these data indicate that minocycline can alleviate cognitive impairment and white matter damage after rUCCAO.

### The early treatment is required for the neuroprotection of minocycline after rUCCAO

**Figure 2:**
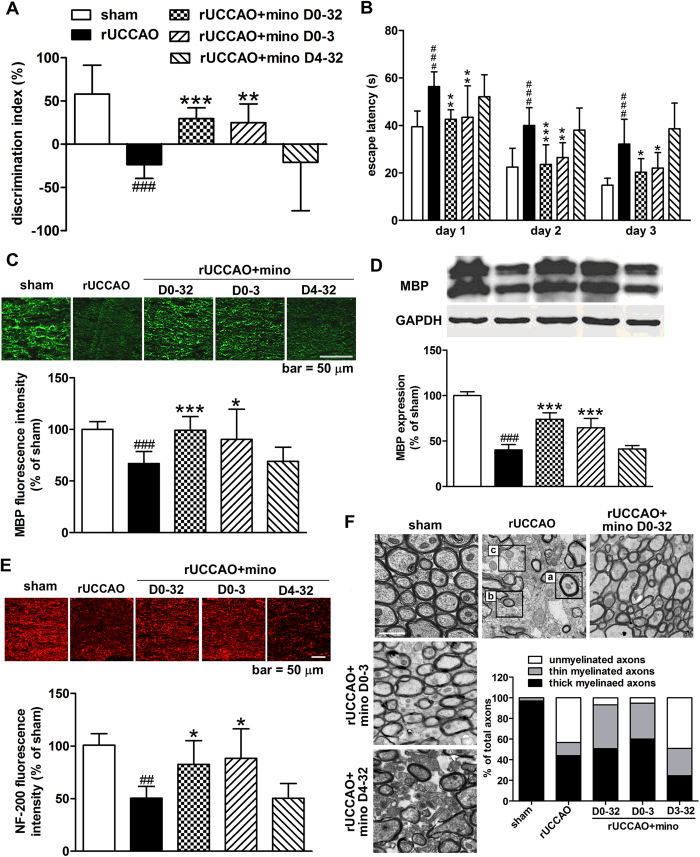
The stage-dependent effect of minocycline (mino) in the object recognition test (**A**) and the Morris water maze test (**B**) under different regimens of administration (D0-32, D0-3 or D4-32) was assessed from 27 to 32 days after rUCCAO. Mice were then sacrificed at 32 days after rUCCAO for immunohistochemistry and electron microscopy examination. The MBP expression (**C**: immunostaining; **D**: western blot) and axonal damage (**E**: NF-200 immunostaining) was assessed. The percentage of thick (a in rUCCAO image), thin myelinated axons (b in rUCCAO image) or unmyelinated (c in rUCCAO image) were quantified from electron microscopy. (**C**,**E**) scale bar, 50 μm; (**F)** scale bar, 1 μm. A, B: n = 9–12; C, E: n = 6–8; D: n = 4–5; F: n = 3. Values are represented as mean ± s.d. ###*P* < 0.001, *vs*. the sham group; *P < 0.05, **P < 0.01, ***P < 0.001, *vs*. the rUCCAO group.

Since the development of white matter damage is a chronic progress, we also want to know the timing of MBP expression decrease after rUCCAO. We found the level of MBP expression in the corpus callosum increased from 1 day to 3 days after rUCCAO, then progressively declined and was greatly lower in mice 7 days after rUCCAO than in the sham group examined by immunofluorescent analysis and Western blot assay (Supplementary Fig. S2). So we hypothesized the action of minocycline may take place at the late stage after rUCCAO. We compared the effects of minocycline (25 mg·kg^−1^d^−1^) administered in the entire course (D0-32), at the early stage (D0-3) or at the late stage (D4-32) after rUCCAO on cognitive ability and white matter damage. Unexpectedly, minocycline administrated at D0-32 and D0-3, but not D4-32, remarkably elevated the discrimination index in the object recognition test (Fig. 2A, *P* < 0.01) and markedly shortened the escape latency in training trials in the Morris water maze test (Fig. 2B; two-way ANOVA indicated a significant difference, *P* < 0.001).

Similarly, minocycline administrated at D0-32 and D0-3 recovered the MBP expression, but minocycline administration at D4-32 had no such effect (Fig. 2C,D). Axons examined by Neurofilament 200 (NF-200) immunostaining showed a pronounced damage after rUCCAO, which was markedly ameliorated by minocycline administrated at D0-32 and D0-3, but not D4-32 (Fig. 2E). To further study the action of minocycline on myelination, the percentages of unmyelinated (G-ratio = 1), thin myelinated (0.8 < G-ratio < 1, often indicating remyelinated axons), and thick myelinated axons (G-ratio ≤ 0.8, often indicating preserved myelinated axons) were quantified with electron microscopy^29^ (Fig. 2F). The number of the unmyelinated axons was greatly increased in the rUCCAO group, while minocycline administrated at D0-32 and D0-3, but not D4-32, dramatically reduced the percentage of unmyelinated axons (*P* < 0.001). Minocycline administrated at D0-32 remarkably increased the percentage of thin myelinated axons (*P* < 0.01), while the treatment at D0-3 greatly elevated the percentage of both the thin and thick myelinated axons (*P* < 0.05). It suggests that early treatment of minocycline alleviated demyelination as well as improved remyelination after rUCCAO. Together, the results suggest that early stage treatment of minocycline is effective in neuroprotection after rUCCAO.

### Minocycline-induced protection is independent of inhibition of microglial activation at the early stage after rUCCAO

**Figure 3:**
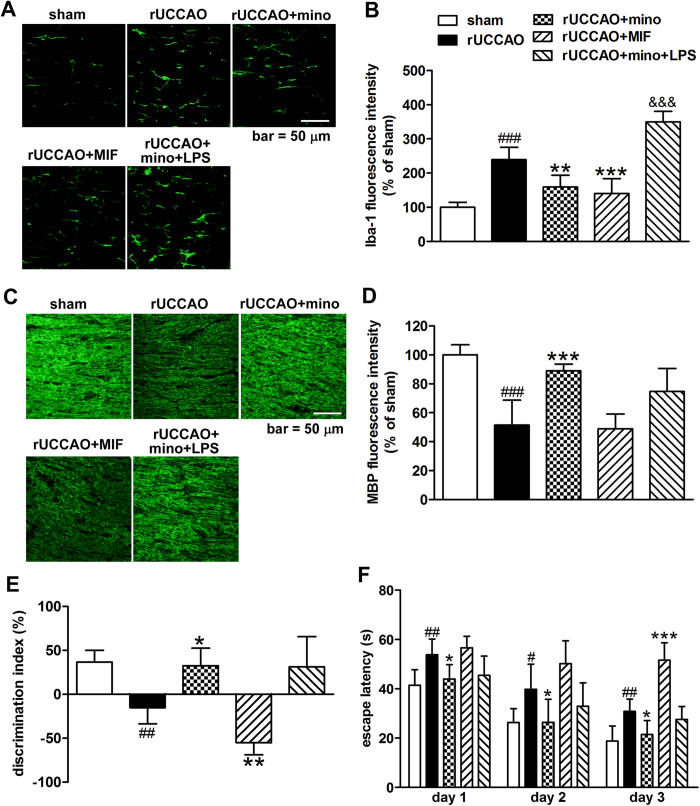
The activation of microglia (B; Iba-1 staining) after minocycline (mino), MIF and LPS treatment was examined at 3 days after rUCCAO, with representative photographs shown in (**A**). The MBP protein expression (**C**: representative photomicrographs, **D**: quantitative analysis) and the cognitive ability of mice tested with the object recognition test (**E**) and the Morris water maze test (**F**), after minocycline (D0-3), MIF and LPS treatment were evaluated at 32 days after rUCCAO. Scale bar, 50 μm. B, D: n = 6–8; E, F: n = 7–8. Values are represented as mean ± s.d. #*P* < 0.05, ##*P* < 0.01, ###*P* < 0.001, *vs*. the sham group; *P < 0.05, **P < 0.01, ***P < 0.001, *vs*. the rUCCAO group; & & & *P* < 0.001, *vs*. the mino group.

Usually the action of minocycline in the brain is related to suppression of microglial activation^16^,^18^,^30^, so we examined whether microglial activation was involved in the action of minocycline. Microglia activation was observed at 3 d after rUCCAO as demonstrated by ionized calcium-binding adapter -1 (Iba-1) immunostaining (Fig. 3A,B). This activation can be sufficiently inhibited by both minocycline and microglia migration inhibition factor (MIF), a specific microglia activation inhibitor^31^. However, we were interested to find that MIF cannot duplicate the effect of minocycline on MBP expression (Fig. 3C,D) and even aggravated cognitive impairment in object recognition test (Fig. 3E) and Morris water maze test (Fig. 3F, two-way ANOVA indicated a significant difference, *P* < 0.001) at 32 d after hypoperfusion. Lipopolysaccharides (LPS) reversed minocycline-induced inhibition of microglia activation through Iba-1 immunostaining. However, LPS had no significant effect on the protection of minocycline regarding MBP expression and cognitive impairment in mice examined with the object recognition test and the Morris water maze test (Fig. 3C–F). These data suggest a different mechanism by which minocycline provides neuroprotection without engaging inhibition of microglial activation at the early stage after rUCCAO.

### Effect of early treatment of minocycline on oligodendrocyte linage cells after rUCCAO

**Figure 4:**
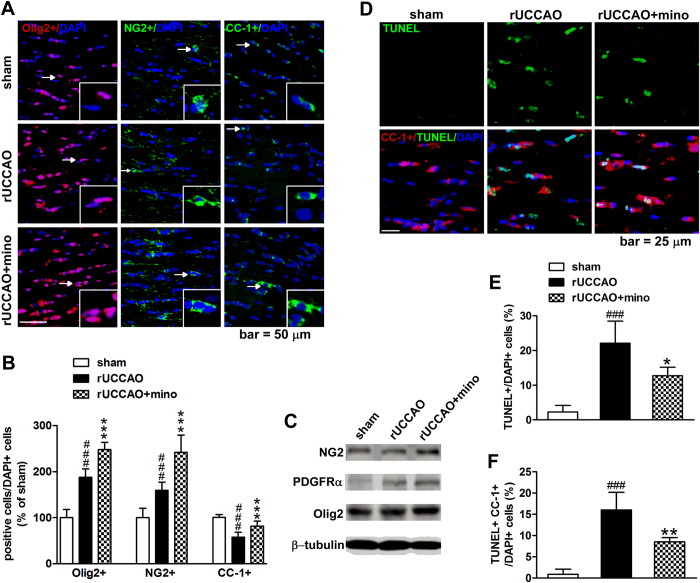
The total oligodendrocytes (Olig2+), OPCs (NG2+) and mature oligodendrocytes (CC1+) after minocycline (mino) D0-3 treatment were calculated as the percentage of the total cells (labeled by DAPI) at 3 days after rUCCAO (**B**), with the representative photomicrographs in (**A**) (the cells with arrows or arrowhead were enlarged in insets). Western blot analysis of NG2+, PDGFRα+ and Olig2 was also performed at 3 days after rUCCAO (**C**). The apoptosis of mature oligodendrocytes was analyzed by TUNEL staining, with representative photomicrographs in (**D**) and the quantitative analyses in (**E** and **F**), which were calculated as the percentage of the total cells (labeled by DAPI). Scale bar: 50 μm in A and 25 μm in C. A, B: n = 6–8; E, F: n = 4. Values are represented as mean ± s.d. ###*P* < 0.001, *vs*. the sham group; *P < 0.05, **P < 0.01, ***P < 0.001, *vs*. the rUCCAO group.

Myelin is made up by oligodendrocytes, which progress through a series of maturation steps to become myelinating oligodendrocytes: (1) OPCs, (2) pre-oligodendrocytes (OLs), (3) immature/premyelinating oligodendrocytes, and (4) mature oligodendrocytes^27^,^28^. We evaluated the oligodendrocyte population on D3 after early treatment of minocycline (Fig. 4A,B). The number of total oligodendrocytes (oligodendrocyte transcription factor2, Olig2 + cells) and OPCs (NG2 chondroitin sulfate proteoglycan, NG2 + cells) was increased on D3 after rUCCAO. Administration of minocycline on D0-3 further increased the number of total oligodendrocytes (*P* < 0.001) and OPCs (*P* < 0.001). Minocycline also increased the expression of Olig2, NG2 and platelet-derived growth factor receptor-alpha (PDGFRα, OPC biomarker) on D3 after rUCCAO, which also suggested the increase of the total oligodendrocyte and OPC population following the early treatment of minocycline (Fig. 4C). For mature oligodendrocyte (adenomatous polyposis coli, CC-1 + cells) population, we found that they declined following rUCCAO (*P* < 0.001), while minocycline treatment increased the number of mature oligodendrocytes (*P* < 0.001). To explore the protection effect of minocycline on oligodendrocytes, the TdT-mediated dUTP nick end labeling (TUNEL) assay was performed. The number of apoptotic cells in the corpus callosum was markedly increased after rUCCAO, which was partly reversed by minocycline (Fig. 4D,E, *P* < 0.05). Furthermore, by counting the TUNEL + CC-1 + double positive cells, we found that most of apoptotic cells in the corpus callosum are mature oligodendrocytes (72.7 ± 4.8% of TUNEL + cells), and minocycline had a prominent protection on these cells (Fig. 4D,F, *P* < 0.01). On the other hand, the number of immature/premyelinating oligodendrocytes (O4 + cells) was decreased after rUCCAO (*P* < 0.001), and minocycline has no effect on it (Supplementary Fig. S3). Therefore, early treatment of minocycline upregulated the number of OPCs and reduced apoptosis of mature oligodendrocytes, that may account for its protection on the white matter.

### Minocycline promotes the proliferation of OPCs after rUCCAO

**Figure 5:**
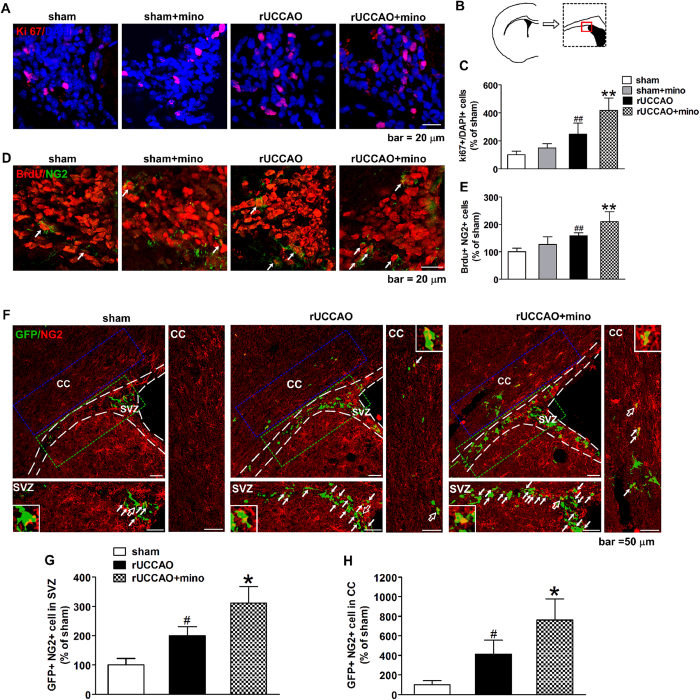
The action of minocycline (mino) on OPCs proliferation in SVZ was determined after rUCCAO. A cartoon depiction shows the analyzed SVZ region (red line) enlarged from the region of a mouse brain with dotted line (**B**). The Ki 67 + cells in SVZ were calculated as the percentage of the total cells (labeled by DAPI, **C**), with representative photomicrographs in A. The BrdU + NG2 + double positive cells (arrows in **D**) in SVZ were counted (**E**). The areas outlined by white dash line in F were SVZ areas, and the SVZ areas with green outline were enlarged in the bottom lanes, while the corpus callosum (CC) areas with blue outline were enlarged in the right lanes. The retrovirus infected (GFP+) and NG2 + double positive OPCs (arrows in F) in SVZ areas shown in the bottom lanes and in CC areas shown in the right lanes were counted (**G**,**H**, the cells with hollow arrows in F are enlarged in insets). (**A**,**D**): scale bar, 20 μm; (**F**): scale bar, 50 μm. (**C**,**E**): n = 5–6; (**G**,**H**): n = 4. Values are represented as mean ± s.d. #*P* < 0.05, ##*P* < 0.01, *vs*. the sham group; *P < 0.05, *P < 0.01, *vs*. the rUCCAO group.

It is well established that OPCs proliferate and differentiate into mature oligodendrocytes in the developing brain^28^, which may also be responsible for the remyelination in a demyelinating lesion. By Ki 67 immunostaining, we found the number of proliferating cells increased in subventricular zone (SVZ) on D3 after rUCCAO, and minocycline further elevated this number (Fig. 5A,C, *P* < 0.01). On the other side, no proliferating cells were observed in the corpus callosum area. By 5’-bromo-20-deoxyuridine(BrdU)/NG2 double immunostaining on D3 after rUCCAO, we found the number of BrdU+ OPCs was greatly increased in mice with rUCCAO compared to that in the sham group, and minocycline treatment on D0-3 remarkably promoted the proliferation of OPCs after rUCCAO (Fig. 5D,E, *P* < 0.01). However, minocycline has no significant effect on the OPC proliferation in sham group from the immunostaining and BrdU/NG2 double immunostaining (Fig. 5A,C–E).

To verify the effect of minocycline on OPC proliferation, a replication-incompetent GFP retrovirus was delivered into the SVZ 3 days before the rUCCAO, which labels local proliferating cells^32^,^33^. Virtually all infected cells were restricted to the SVZ, most of which are also BrdU positive cells (Supplementary Fig. S4). By immunostaining with NG2, we found the proliferating OPCs increased in the SVZ at 3 days after rUCCAO, and minocycline further expanded this OPC pool (Fig. 5F,G, *P* < 0.05). The retrovirus-infected OPCs were also present in the corpus callosum after rUCCAO, and these cells were increased in the minocycline-treated group (Fig. 5F,H, *P* < 0.05). These data suggest that minocycline promotes the proliferation of OPCs at early stage after rUCCAO, which can replenish the OPC pool in the white matter.

### Minocycline promotes the proliferation of OPCs *in vitro*

**Figure 6:**
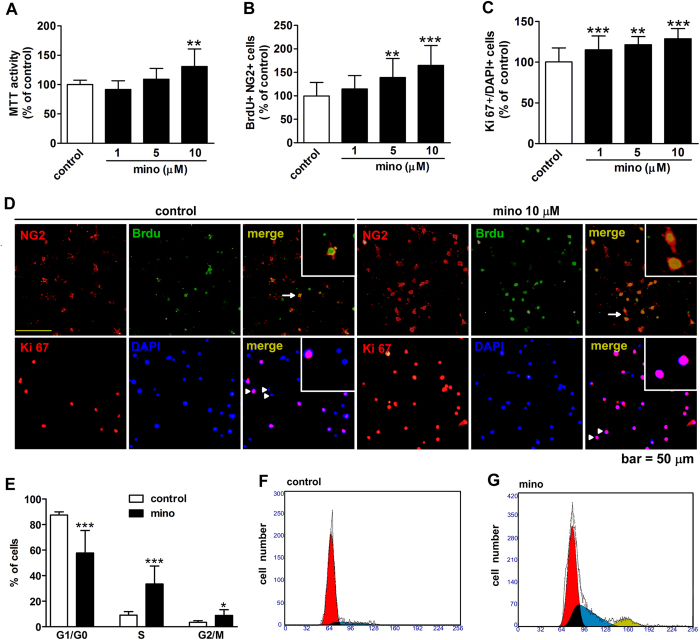
The action of minocycline (mino) on OPCs proliferation was determined in cultured OPCs. The MTT assay was performed after minocycline treatment (**A**). BrdU + cells were calculated as the percentage of NG2 + cells (**B**), and Ki 67 + cells were calculated as the percentage of total cells (labeled by DAPI, **C**), with the representative photomicrographs in (**D**) (the cells with arrows or arrowhead were enlarged in insets). The effect of minocycline (mino, 10 μM) on the cell cycle in cultured OPCs was determined by flow cytometry (**E**), with representative histogram shown in (**F** and **G**). Populations in the G0/G1, S, and G2/M phases are shaded in yellow, red, and blue, respectively. Scale bar, 50 μm. Values are from 3 to 4 independent experiments and represented as mean ± s.d. *P < 0.05, **P < 0.01, ***P < 0.001, *vs*. the control group.

To further confirm the action of minocycline on OPC proliferation, we investigated the effect of minocycline on cultured OPCs. 3-(4, 5)-Dimethythiahiazo (-z-y1)-3.5-diphenytetrazoliumromide (MTT) assay showed that 10 μM minocycline increased the proliferation of OPCs (Fig. 6A). By BrdU or Ki 67 immunostaining (Fig. 6B–D), we found minocycline dose-dependently elevated the number of BrdU+ or Ki 67 + OPCs. We further studied the enhanced OPC proliferation by minocycline *via* flow cytometry. We found minocycline (10 μM) strikingly increased the percentage of OPCs from 9.2% to 33.4% in the S phase, increased the percentage of OPCs in the G2/M phase, and reduced the percentage of OPCs in the G0/G1 phase (Fig. 6E–G). These data suggest that minocycline may facilitate the cell cycle progression into the S and G2/M phases to promote the proliferation of OPCs.

## Discussion

Although the neuroprotection of minocycline *in vivo* and *in vitro* has been addressed^16^,^30^,^34^, contradictory reports still exist^19^,^20^,^35^. In the present study, we demonstrated the protective effects of minocycline under the chronic cerebral hypoperfusion induced by rUCCAO, which well mimics the white matter and cognitive impairments of SIVD^23^,^24^. We found that early treatment of minocycline remarkably ameliorated the cognitive impairment, white matter rarefaction, axonal damage and demyelination, but improved remyelination in the corpus callosum after rUCCAO. Since minocycline is used as an antibiotic in the clinical setting, its safety for human use has been extensively evaluated. Also because it can easily cross the blood-brain barrier, the neuroprotective effect of minocycline in the rUCCAO model makes it a potential therapeutic treatment for SIVD.

The protection of minocycline on hippocampus and white matter after chronic cerebral hypoperfusion has been reported under an entire course treatment^18^,^36^,^37^. However, the overt demyelination takes place at the late stage after hypoperfusion^22^ (Supplementary Fig. S2), therefore the temporal effects of minocycline were investigated after hypoperfusion. We found that minocycline treated at the early stage (day 0-3), but not the late stage (day 4–32), provided the neuroprotection comparable to the full-course treatment, for the treatment of on D0-3 markedly reversed cognitive impairment and attenuated the white matter damage. Our data suggest that the administration time window of minocycline is crucial for treating SIVD, as early treatment is necessary while late treatment may be dispensable. A recent study showed that minocycline reduced the volume of injury at 24 h but not 7 d after transient MCAO, implicating minocycline provides an early but transient protection^38^. Here, we showed that the early transient treatment of minocycline displayed a prominent neuroprotection at the late stage after rUCCAO, so the therapeutic strategy of early treatment is sufficient to provide a long-term effective protection. This time sensitive neuroprotection also hints that other pharmacological treatments for SIVD should be revisited for the best administration time window.

Usually, hypertension, diabetes mellitus, hyperlipidemia and aging are the high risk factors for SIVD^39^,^40^. A decrease in the cerebral blood flow in these patients examined by a cerebral perfusion scan may predict the occurrence of SIVD. Moreover, an increase in the intensity of MBP expression was observed at the early stage after rUCCAO (Supplementary Fig. S2) and this increase was possibly resulted from a reactive change of myelination. Also, the myelin change may serve as a surrogate marker for the early diagnosis with MRI. Therefore, monitoring these patients and timely administering minocycline at very early stage of SIVD may be a preferable treatment approach for such a condition.

The inconsistent timing between the early minocycline treatment and the late stage demyelination makes it intriguing to investigate the mechanism of the minocycline-induced neuroprotection. Though the pathogenesis of the white matter damage in SIVD is still unclear, the white matter damage frequently coincides with myelin degradation and the differentiating status of oligodendrocytes. Oligodendrocytes, myelin-forming glial cells of the central nervous system, are known to be quite vulnerable to ischemic stress, as ischemia or hypoxia causes loss of myelin^41^,^42^. However, if OPCs proliferated and differentiated into oligodendrocytes, remyelination could be expected^43^. In the present study, minocycline partly alleviated the loss of mature oligodendrocytes at the early stage after rUCCAO, which may account for its protection against demyelination later on. Furthermore, we found minocycline promoted the OPC proliferation from both *in vivo* and *in vitro* observations. The increase of OPC proliferation may enlarge the regeneration population of oligodendrocytes, so as to benefit the remyelination process^44^. Thereafter, the demyelinated axons could undergo ensheathment with new myelin sheaths, which can lead to functional recovery^32^,^45^,^46^.

The direct effect of minocycline on oligodendrocyte differentiation was not observed (Supplementary Fig. S3). However, the proliferating OPCs may migrate to the corpus callosum and differentiate into mature oligodendrocytes, which is proposed based on the following evidence: 1) minocycline increased the number of OPCs in the corpus callosum (Fig. 4A,B), but no proliferating cells were observed there; 2) more proliferating OPCs infected by the retrovirus in the SVZ were found in the corpus callosum in minocycline-treated group (Fig. 5F,H); 3) the number of O4 + cells were not changed on D3 but increased on D7 after D 0-3 treatment of minocycline (Supplementary Fig. S3); 4) eventually, remyelination in the minocycline-treated group was more prominent than that in the control group, due to an increase in the percentage of the thin myelinated axons (Fig. 2F). Taken together, the protection of minocycline at the early stage after rUCCAO may be contributed by the promotion of OPC proliferation and the protection of mature oligodendrocytes.

Previous studies suggest that minocycline-mediated suppression of microglial activation is likely to contribute to neuroprotection^16,30^. After chronic cerebral hypoperfusion resulted from bilateral permanent occlusion of the common carotid arteries in rats, minocycline was found to alleviate white matter damage together with inhibiting microglia activation^18^. However, there is still lack of direct evidence for the involvement of the inhibition of microglial activation in the protection of minocycline after cerebral chronic hypoperfusion. In this study, we were surprised to find that MIF, a specific microglia activation inhibitor, showed different action on cognitive impairment and MBP expression after rUCCAO compared with minocycline treatment. Moreover, although LPS reversed the inhibition of microglia activation conferred by minocycline, it has no effect on the protection of minocycline regarding cognitive impairment and MBP expression following rUCCAO. It suggests that the neuroprotection of minocycline at the early stage after rUCCAO is very likely not related to the microglia activation, so we propose that it may be due to the action of minocycline on OPC proliferation, besides its protection on mature oligodendrocytes.

Indeed, we found the number of OPCs in the corpus callosum was not increased in the MIF group, and LPS did not affect the OPC pool when co-treated with minocycline after rUCCAO (Supplementary Fig. S5). Schmitz *et al.*^43^ reported that minocycline improves survival and restores maturation of OPCs after oxygen-glucose deprivation (OGD). In our *in vitro* experiments, minocycline promoted the proliferation of OPCs as revealed by the MTT assay and BrdU and Ki 67 staining, which is possibly due to more OPCs entering the S phase and the subsequent G2/M phase in cultured OPCs. In other cases, the expression of soluble molecules from microglia are often suggested to be reason for the protection of minocycline, such as in neonatal rats following hypoxic-ischemic exposure or in rats after transient focal cerebral ischemia^17^,^30^. In the cuprizone-induced demyelination, minocycline even reduces remyelination by suppressing ciliary neurotrophic factor expression from microglia^35^. Our data highlighted a microglia-independent protection of minocycline probably through promoting OPC proliferation, which may result from its action of attenuating injury or improving various pathways of repair in the brain after rUCCAO. Indeed, many studies have suggested the anti-oxidant and anti-apoptosis activities of minocycline^47^, that may contribute to its promotive effect on OPC proliferation under the cerebral chronic hypoperfusion *in vivo* or oxygen toxicity from *in vitro* culture^48^. These studies at least suggested that minocycline may exhibit variable actions related or not related to microglia under different insults, and the stage related study here makes it easy to understand the effects and underlying mechanism of minocycline.

In conclusion, we demonstrated that early treatment is critical for minocycline to alleviate white matter and cognitive impairments after chronic cerebral hypoperfusion. The protection of minocycline may result from its robust effects on OPC proliferation and mature oligodendrocyte loss, but not the inhibition of microglia activation. So, early therapeutic time window may be crucial for its application in the treatment of SIVD.

## Methods

### Animal studies

Male 8–9 weeks old C57BL/6 mice were used. Mice were kept under standard animal housing conditions (12  h light/dark a cycle) with food and water *ad libitum*. All experiments were approved by the Zhejiang University Animal Experimentation Committee and were in complete compliance with the National Institutes of Health Guide for the Care and Use of Laboratory Animals. Efforts were made to minimize any pain or discomfort, and the minimum number of animals was used.

Mice were randomized into sham or different treatment groups. Mice were anesthetized with sodium pentobarbital (60 mg·kg^−1^, i.p.). The right common carotid artery was isolated from the adjacent vagus nerve and double-ligated with 6-0 silk sutures to perform rUCCAO. Sham-operated mice were subjected to the same procedure, except for the carotid ligation. After rUCCAO, mice were intraperitoneally injected with minocycline (Sigma, USA) every day for the following several regimens: D0-32 (day 0 to day 32, 10 or 25 mg·kg^−1^), D0-3 (day 0 to day 3, 25 mg·kg^−1^) and D4-32 (day 4 to day 32, 25 mg·kg^−1^). For MIF (Bachem, Switzerland) or LPS (Sigma, USA) injection, mice were injected with MIF (1 μM·μl^−1^, 1μl, i.c.v.) or LPS (100 ng·μl^−1^, 1 μl, i.c.v.) immediately after rUCCAO. To study cell proliferation, mice were injected with BrdU (Sigma, USA; 50 mg·kg^−1^ i.p.) twice per day for 3 consecutive days after rUCCAO, and were sacrificed 2 h after the last injection on D3. On D27 after rUCCAO, mice were subjected to the object recognition test, as a pre-test, and on D28 the same test was used for the test procedure. Morris water maze, testing commenced on D29 and continued for four days. On D3 or D32 post-rUCCAO, mice were sacrificed and brain tissues were removed for histochemical staining or Western blot assay.

### Object recognition test

The object recognition test was performed to evaluate non-spatial working memory related to the frontal-subcortical circuits^23^. A glass box (30 × 45 × 30 cm) was used. The objects to be discriminated were cubes (green), pyramids (blue), and cylinders (red) of 5.8 cm high. On the first day of the test, mice were allowed to explore the box without any objects for 10 min. On the second day of the test, there were two trials with an intertrial interval of 1 h. In the first trial, two identical objects were presented on two opposite sides of the box, and a mouse was placed in the box and allowed to explore for 10 min. During the second trial, one of the objects presented in the first trial was replaced with a new object and the mouse was allowed to explore for 3 min. Object exploration was considered only if the mouse is directing the nose at a distance <1 cm from the object and/or touching it with the nose. The exploration time spent on each of the familiar (F) object and the new (N) object was recorded manually. The discrimination index was calculated by (N − F/N + F) × 100% for intergroup comparisons.

### Morris water maze test

The Morris water maze is currently the most frequently used paradigm to evaluate cognitive abilities in mice^23^,^26^. A circular pool, with a diameter of 150 cm and a height of 50 cm, was filled with water at a depth of 30 cm. The hidden platform was submerged 1.5 cm below the surface of the water, and the mice were placed randomly between the quadrants in the water. Our experimental design was 4 trials for the first day and 6 trials for the next two days for the acquisition phase, and the interval between the trials was 10 min. During each trial, the escape latency was defined as the time to reach the platform and climb up out of the water, but limited to 60 s. The average escape latency for each day was calculated.

### Histochemical staining

Mice were perfused transcardially with 4% paraformaldehyde in 0.1 M phosphate buffer (PBS, pH 7.4). Brains were removed and post-fixed in 4% paraformaldehyde at 4 °C for 24 h, and then in 30% sucrose in PBS for 3d. Frozen coronal brain sections (10 μm thick) were cut on a cryostat (SM2000R; LEICA, Germany). For immunostaining, individual brain sections were incubated with 3% normal donkey serum in PBS containing 0.3% Triton X-100 for 2 h. The tissue sections for BrdU incorporation analysis were pretreated with 2 N HCl for 30 min, then incubated with the following appropriate primary antibodies overnight: rabbit anti-Iba1 (1:1000; Wako, Japan); rat anti-MBP (1:250; Millipore, USA); mouse anti-NF 200 (1:300; Abcam, USA); rabbit anti-Olig2 (1:500; Millipore, USA); mouse anti-CC-1 (1:50; Abcam, UK); rabbit anti-NG2 (1:50; Millipore, USA); rabbit anti-Ki 67 (1:200; Abcam; UK); mouse anti-BrdU (1:1000; Sigma, USA). After being washed in PBS, sections were incubated with appropriate secondary antibodies for 2 h at room temperature. For double labeling of TUNEL and CC-1, after the sections were incubated with secondary antibody, an *in situ* cell death detection kit (Roche, USA) was used to visualize TUNEL-labeled nuclei. The sections were observed under a fluorescence microscope (Olympus, Tokyo, Japan) or confocal microscope (Olympus, Tokyo, Japan). The fluorescence intensity and cell counting were examined by Image J software (NIH, MD, USA) in the ipsilateral corpus callosum from three slices at the similar coronal position of each animal. CC-1+, Olig2+, NG2+, TUNEL+ or TUNEL + CC-1 + double positive cells were calculated as a percentage to the total cells (labeled by DAPI). In the ipsilateral SVZ, the Ki 67 + cells were calculated as a percentage to the total cells (labeled by DAPI); BrdU + NG2 + double positive cells were counted from 4 slices at the similar coronal position (AP = 0.5 mm) of each mouse. The percentages or the cell numbers were then corrected by the analyzed size and normalized to those found in the sham groups.

### Retroviral injection

New born cells in the SVZ were traced with a pROV-EF1a-GFP retrovirus^33^ (Neuron Biotech, China) by direct injection of the virus in the SVZ (AP = 0.5 mm, RL = 1.5 mm, H = −2.5 mm). Viral stock of 1 μl was injected (titer 1 × 10^8^ cfu/ ml) in 8 min at 3 days before rUCCAO. Mice were sacrificed at 3 days after rUCCAO, and coronal tissue sections (30 μm thick) were obtained. Z-stacks of 2 μm-thick single-plane images over the entire slice were captured using a confocal microscope (Olympus, Tokyo, Japan). Four consecutive slices at the similar coronal position (AP = 0.5 mm) were taken for observation from each animal. Viral infected GFP^+^ cells, which were also immunostained with NG2^+^, were counted in the ipsilateral corpus callosum and SVZ. The cell numbers were then corrected by the analyzed size and normalized to those found in the sham groups.

### Electron microscopy

Mice were transcardially perfused with a fixation buffer containing 2.5% glutaraldehyde and 4% paraformaldehyde. Samples of the corpus callosum were obtained and kept in the same buffer for 48 h at 4 °C. After that, samples were postfixed in 1% osmium tetroxide, dehydrated in cold ethanol, and infiltrated and embedded in Epon812. Sections of 120 nm were stained with uranyl acetate and lead citrate. The grids were then imaged using a transmission electron microscope. Quantification of myelinated axons was performed on four 8300× images per animal. G ratios (the ratio of axon diameter to the axon plus myelin sheath diameter) were calculated using the Image J software for at least 150 fibers per animal.

### Western blots

The corpus callosum was dissected and homogenized in an ice-cold lysis buffer. Western blot was carried out by standard protocol with proper antibodies like mouse anti-glyceraldehyde-3-hposphate dehydrogenase (anti-GAPDH; 1:5000; Kang-chen, China), rat anti-PDGFRα (1:500; Abcam, USA ); rabbit anti-Olig2 (1:500; Millipore, USA); rabbit anti-NG2 (1:500; Millipore, USA) or rat anti-MBP (1:500; Millipore, USA) overnight at 4 °C. Membranes were washed three times with TBST buffer and incubated with IRDye 800 anti-rabbit Molecular Probe (1:8000; LI-COR Biosciences, USA) or IRDye 700 anti-mouse/rat Molecular Probe (1:3000; LI-COR Biosciences, USA) for 2 h at room temperature. Images were acquired with the Odyssey infrared imaging system and analyzed by the Odyssey software^49^.

### Primary cortical OPC culture

Primary cortical OPC cultures were prepared from postnatal D2 Sprague-Dawley rats, as described previously^50^, which were purchased from Zhejiang Academy of Medical Science. The OPCs were cultured in the Neurobasal Medium (Gibco, USA) supplemented with the platelet-derived growth factor (PDGF-AA 10 ng·ml^−1^; PeproTech, USA) and the basic fibroblast growth factor (bFGF; 10 ng·ml^−1^; PeproTech, USA) for 3 days. More than 95% of the cultured cells were OPCs as identified by immunofluorescent staining for NG2. On the third day, OPCs were treated with minocycline (1, 5 or 10 μM) for 24 h. The proliferation status of OPCs was determined by MTT assay^51^ or BrdU (10 μM BrdU given 24 h before assay) and Ki 67 immunostaining. For immunostaining, cells were fixed in 4% paraformaldehyde in PBS for 15 min, blocked with PBS containing 3% normal donkey serum and 0.3% Triton X-100 for 20 min. For BrdU staining, cells were then treated with 2 N HCl for 30 min. Primary antibodies (anti-BrdU; 1:1000; Sigma, USA or anti-Ki 67; 1:500; Millipore; USA) were applied at 4 °C for 24 h. The cells were washed in PBS and incubated with Alexa conjugated secondary antibodies for 2 h at room temperature. BrdU + cells were calculated as a percentage to total NG2 + cells, and Ki 67 + cells were calculated as a percentage to total cells (labeled by DAPI). These percentages were then normalized to those found in the sham groups.

### Flow cytometry

For OPC cell cycle analysis , OPCs were treated with minocycline (10 μM) for 24 h, detached with trypsin and washed with PBS for three times. The cells were kept in ice-cold 70% EtOH at 4 °C overnight and then incubated with propidium iodide (PI, 50 μg·ml^−1^) and RNase 20 (μg·ml^−1^), followed by incubation in the dark for 30 min at 37 °C. A total of 20,000 cells from each treatment were counted. The cell cycle was determined by the Cytomics FC 500 MCL (Beckman-Coulter, CA, USA). The ratios of the cells in G0/G1, S and G2/M phases were calculated.

### Statistical analysis

All data were collected and analyzed in a blind fashion. Data were presented as mean ± standard deviation (s.d.). Statistical analyses were done with SPSS 11.5 for Windows or GraphPad Prism 5.0. One-way analysis of variance (ANOVA) followed by the Tukey’s *post-hoc* test was used for multiple comparisons. The escape latency in the Morris water maze test was analyzed by two-way ANOVA followed by the LSD test.

## Additional Information

**How to cite this article**: Ma, J. *et al.* Early treatment of minocycline alleviates white matter and cognitive impairments after chronic cerebral hypoperfusion. *Sci. Rep.* **5**, 12079; doi: 10.1038/srep12079 (2015).

## Supplementary Material

Supplementary Information
