# Supplementary material for: Early treatment of minocycline alleviates white matter and cognitive impairments after chronic cerebral hypoperfusion
Source: Sci Rep. 2015 Jul 15;5:12079. doi: 10.1038/srep12079 (PMC4502604; doi:10.1038/srep12079)
Supplement: Supplementary Information [file srep12079-s1.pdf]

Supplementary information

Early treatment of minocycline alleviates white matter and cognitive impairments after chronic cerebral hypoperfusion

Jing Ma[a,c,1], Jing Zhang[a,1], Wei Wei Hou[a,1], Xiao Hua Wu[a], Ru Jia Liao[a], Ying Chen[a], Zhe Wang[a], Xiang Nan Zhang[a,b], Li San Zhang[a], Yu Dong Zhou[a], Zhong Chen[a,b], Wei Wei Hu[a,b*]

[a]Department of Pharmacology, Key Laboratory of Medical Neurobiology of the Ministry of Health of China, College of Pharmaceutical Sciences, Zhejiang University, Hangzhou, Zhejiang 310058, P. R. China; [b]Collaborative Innovation Center for Diagnosis and Treatment of Infectious Diseases, Hangzhou, Zhejiang 310003, P. R.China; [c]Department of Pharmacy, Xinhua Hospital, Shanghai Jiaotong University School of Medicine, Shanghai 200092, P. R. China

[*]Correspondence to:

Wei-wei Hu, Ph.D., E-mail address: huww@zju.edu.cn;

Department of Pharmacology, Key Laboratory of Medical Neurobiology of the Ministry of Health of China, College of Pharmaceutical Sciences, Zhejiang University, Hangzhou, 310058, China.

Tel: +86-571-88208227; Fax: +86-571-88208228.

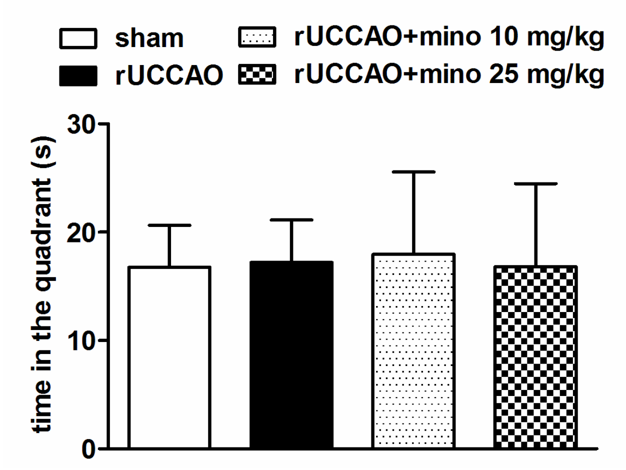

Figure S1. The effect of minocycline (mino) in the probe trail of Morris water maze test at 32 days after rUCCAO. n = 8-9. Values are represented as mean ± s.d.

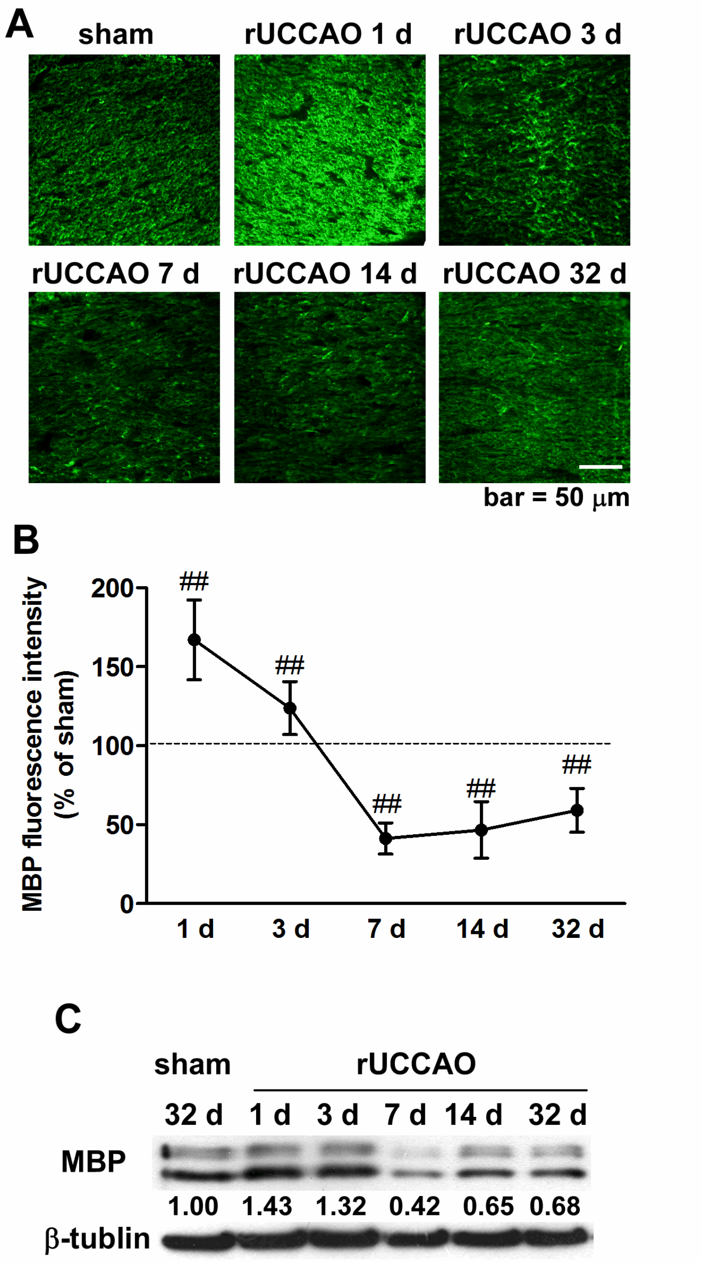

Figure S2. Representative photomicrographs of MBP expression by immunostaining in corpus callosum at different time after rUCCAO are shown in A and the quantitative analyses are shown in B. n = 7-8. Values are represented as mean ± s.d. Scale bar, 50 μm. Representative western blots showed MBP expression in corpus callosum after rUCCAO in C. The digits below represent the semiquantified optidensity of the bands from at least three samples, and the control bands were defined as 1.00. ##*P* <0.01, *vs*. the sham group.

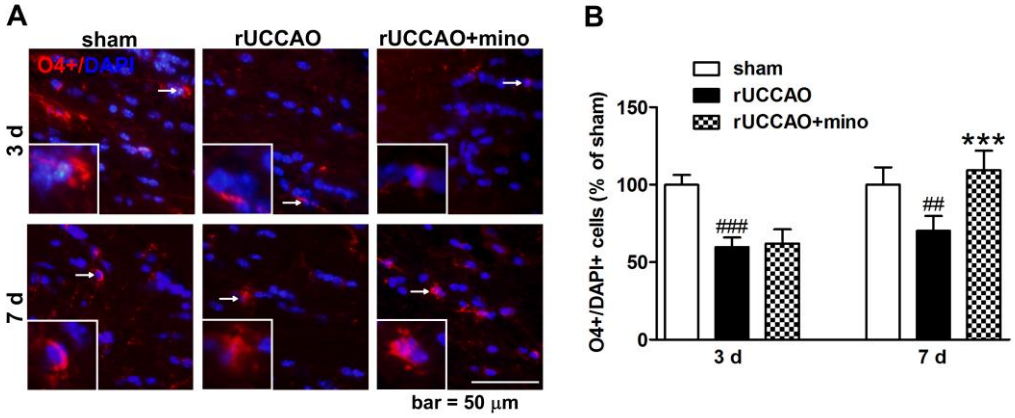

Figure S3. The change of premyelinating oligodendrocytes (O4+) after minocycline (mino) D0-3 treatment was examined at 3 and 7 days after rUCCAO.
Brain sections were incubated with 3% normal donkey serum in PBS containing 0.3% Triton X-100 for 2 h, and incubated with mouse anti-O4 Antibody (clone 81; 1:50; Millipore, USA) at 4 ℃ overnight. After repeated wash in PBS, slices were incubated Alexa 594 conjugated anti-mouse IgG (1:400; Invitrogen, CA) for 2 h at room temperature. The sections were then washed and mounted in mounting media containing 4',6-diamidino-2-phenylindole (DAPI; 1:1000; Sigma, USA). The cell counting was examined by Image J software (NIH, Bethesda, MD) in five areas of the corpus callosum from three slices at the same position of per animal. O4+ cells were calculated as the percentage of the total cells (labeled by DAPI). The percentages were then normalized to sham group. The representative photomicrographs are shown in A, and the quantitative analyses are shown in B. The cells with arrows were enlarged in insets. Scale bar, 50 μm. n=4. Values are represented as mean ± s.d. ##*P* <0.01, ###*P* <0.001, *vs*. the sham group; ****P* < 0.001 *vs*. the rUCCAO group.

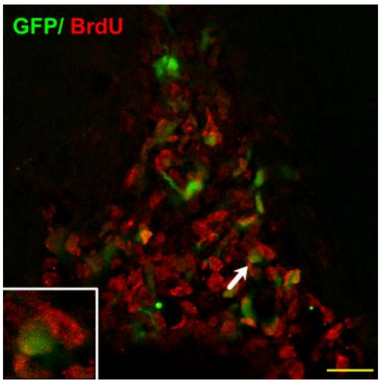

Figure S4. GFP+ retrovirus-infected cells in SVZ were stained with BrdU at 3 days after the injection of GFP+ retrovirus. A pROV-EF1a-GFP retrovirus (Neuron Biotech, China, titer 1x10^8 cfu/ml, 1 μl) was injected into the SVZ (AP= 0.5 mm, RL=1.5 mm, H = -2.5 mm). Mice were then given BrdU 50 mg/kg twice a day for consecutive 3 days, and sacrificed thereafter. Coronal tissue sections showed that 86.9±4.2% GFP+ cells

were double labeled by BrdU (n = 3). The representative photomicrograph of SVZ is shown, and the GFP+ and BrdU double positive cell with arrow is enlarged in the inset. Scale bar, 20 μm.

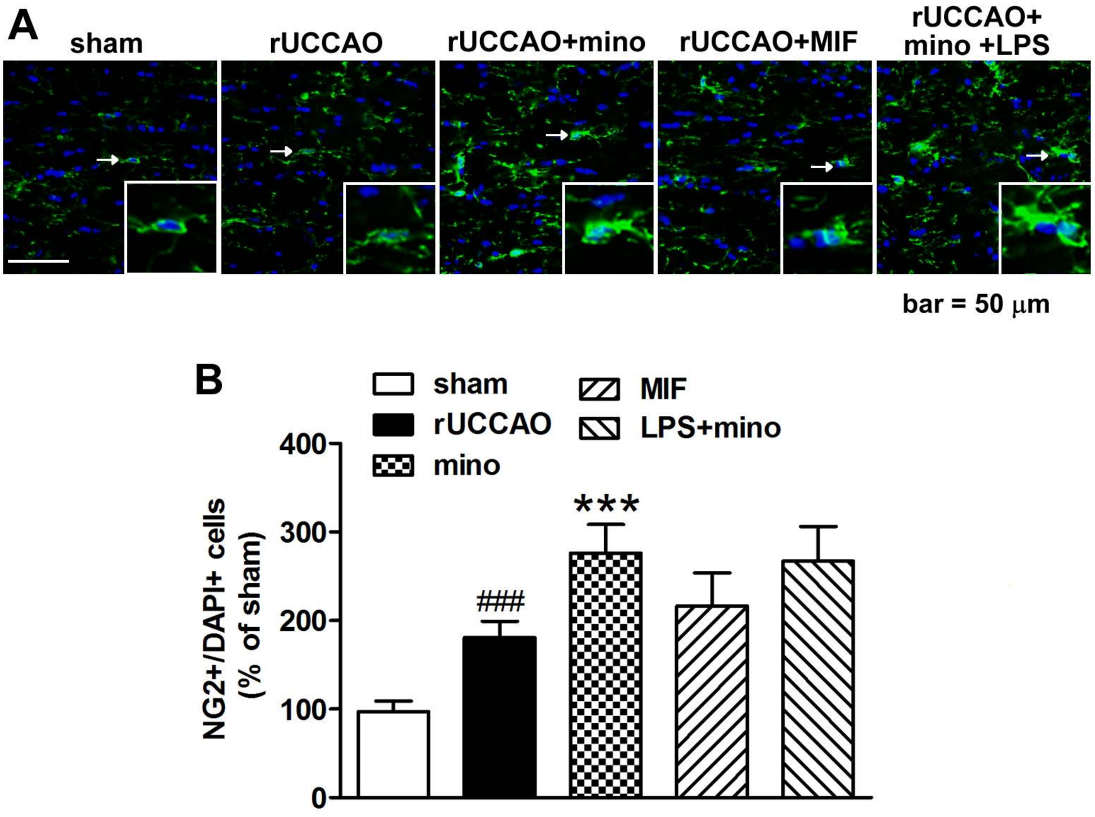

Figure S5. The OPCs (NG2+) after minocycline (mino) D0-3, MIF and LPS treatment was calculated as the percentage of the total cells (labeled by DAPI) at 3 days after rUCCAO (B), with representative photographs in A. The cells with arrows were enlarged in insets. Scale bar, 50 μm. n = 4-6. Values are represented as mean ± s.d. ###*P* <0.001, *vs*. the sham group; ****P* <0.001, *vs*. the rUCCAO group.
